# Consequence of Indoor Air Pollution in Rural Area of Nepal: A Simplified Measurement Approach

**DOI:** 10.3389/fpubh.2015.00005

**Published:** 2015-01-26

**Authors:** Chhabi Lal Ranabhat, Chun-Bae Kim, Chang-Soo Kim, Nilambar Jha, K. C. Deepak, Fredric A. Connel

**Affiliations:** ^1^Department of Preventive Medicine, Wonju College of Medicine Yonsei University, Wonju, Gangwon, South Korea; ^2^Institute for Poverty Alleviation and International Development, Yonsei University, Wonju, Gangwon, South Korea; ^3^BP Koirala Institute of Health Science, School of Public Health and Community Medicine, Dharan, Nepal; ^4^Department of Health Services, School of Public Health, University of Washington, Seattle, WA, USA

**Keywords:** indoor air pollution, biomass, acute respiratory infection, health problems, cooking fuel

## Abstract

People of developing countries especially from rural area are commonly exposed to high levels of household pollution for 3–7 h daily using biomass in their kitchen. Such biomass produces harmful smoke and makes indoor air pollution (IAP). Community-based cross-sectional study was performed to identify effects of IAP by simplified measurement approach in Sunsari District of Nepal. Representative samples of 157 housewives from household, involving more than 5 years in kitchen were included by cluster sampling. Data were analyzed by SPSS and logistic regression was applied for the statistical test. Most (87.3%) housewives used biomass as a cooking fuel. Tearing of eyes, difficulty in breathing, and productive cough were the main reported health problems and traditional mud stoves and use of unrefined biomass were statistically significant (*p* < 0.05) and more risk (AOR > 2) with health problems related to IAP. The treatment cost and episodes of acute respiratory infection was >2 folders higher in severe IAP than mild IAP. Simplified measurement approach could be helpful to measure IAP in rural area. Some effective intervention is suggested to reduce the severe level of IAP considering women and children.

## Introduction

Indoor air pollution (IAP) can be traced to prehistoric times when humans first moved to temperate climates and it became necessary to construct shelters and use fire inside them for cooking, warmth, and light. Fire led to exposure to high levels of pollution, as evidenced by the soot found in prehistoric caves (1). Approximately half of the world’s population and up to 90% of rural households in the developing countries still rely on unprocessed biomass fuels in the form of wood, dung, and crop residues (2). People in developing countries are commonly exposed to high levels of pollution for 3–7 h daily over many years (3). During winter in cold and mountainous areas, exposure may occur over a substantial portion of each 24-h period (4). Because of their customary involvement in cooking, especially women’s exposure is much higher than men’s (5). Young children are often carried on their mothers’ backs while cooking is in progress and therefore spend many hours breathing smoke (1).

Many of the substances in biomass smoke can damage human health. The most important (substances) are particles, carbon monoxide, nitrous oxides, sulfur oxides (principally from coal), formaldehyde, and polycyclic organic matter, including carcinogens such as benzopyrene (6). Particles with diameters below 10 μ (PM_10_), and particularly those <2.5 μ in diameter (PM_2.5_), can penetrate deeply into the lungs and appear to have the greatest potential for damaging health (7). The pollutants produced by unprocessed fuel are carbon monoxide, sulfur oxide, particles, and volatile organic, which are responsible to produce different disease and illness in human beings (8). The pollutants from biomass increase the risk of acute respiratory infections (ARIs) in childhood, chronic obstructive pulmonary disease (COPD) and lung cancer in adult (9, 10). It is the most important cause of death for children under five in developing countries. Evidence also exists of associations with low birth weight (LBW), increased infant and perinatal mortality, pulmonary tuberculosis, nasopharyngeal and laryngeal cancer, cataract, and, specifically in respect of the exposure of coal, with lung cancer to the non-smoker too (1, 11). Biomass users illustrated high suppression in the total number of T-helper (CD4+) cells and B (CD19+) cells, while appreciable rise was documented in the number of CD8+ T-cytotoxic cells and CD16+CD56+ natural killer (NK) cells and consistent finding among biomass users was rise in regulatory T (Treg) cells (12). It proved that IAP is not only arising the health problems but also responsible to worsen the other disease and illness by reducing the immunity.

Nepal is the country of village where >80% live in villages and 75% population are using the unprocessed biomass like firewood, animal dung, and some paper residue leaves of trees as a kitchen fuel (13). The housing structure is very vulnerable for IAP because 70% households have wooden and mud bonded house with poor ventilation (14). Here, the majority of populations live in countryside and they used unprocessed biomass as a cooking fuel. As a result, the situation of ARI and skin diseases has been first and second in morbidity rank each year. In addition, the COPDs, eye problems, and other cough related diseases are frequently recurrent disease. So, there could be some association with the housing condition and IAP (15).

To study the magnitude of harmful gas like carbon dioxide, carbon monoxide, nitrogen oxide, benzene butadiene, formaldehyde, etc., there is necessary to measure the pollutants by some sophisticated equipment at the level of >10 p.m. Such approach can precise the level of IAP produced from different sources, but they are quite unpractical in a rural context for the household and the researchers. There is still lacking to measure the indoor air pollutants by simple methods and arising health problems due to the long exposure in rural areas and developing countries. The simplified IAP measurement questionnaire was prepared based on Warwick H. study (16) related to type of stoves, type of fuel, used in kitchen, smoking situation in family members, and some behavioral pattern of cooking member than any devices. The main objective of this research is to explore the health consequence related to IAP and its major effects as well as the relation of some factors in community level without sophisticated measurement of the pollutants and clinical diagnosis of the health problems.

## Methodology

### Study population and sampling

The study was a cross-sectional study conducted in Khanar village development committee (VDC); a grass root level political division in Sunsari District of Nepal in 2013. The VDC was selected purposively. Cluster sampling method was used to involve all kinds of geographical area. The sample size was calculated on the basis of proportion of using biomass (72.3%) (14) as 157 households and the housewives were included in the study who are involving in their kitchen more than 5 years. The sample size were calculated on Cochran equation (17) where the sample size would be: *Z* denotes the abscissa of the normal curve that cuts off an area α at the both tails (1 − α equals the desired confidence level, e.g., 95%), *e* is the desired level of precision (0.07), *p* is the estimated proportion (0.72) of an attribute that is present in the population, and *q* is 1−*p* which is 0.28.

### Data collection and instruments

Semi-structure interview, observation, and focus group discussion methods were used to collect information. The questionnaire, observation checklist, and interview guideline were the tool and translated in Nepali language. Questionnaires were pre-tested in Tarahara VDC. After the pre-test in 20 households, we modified the observation checklist about the kitchen situation. The field researchers were selected based on their previous experiences, familiarity with study areas, local language, and culture and they were provided intensive training to get the adequate information and minimizing the errors.

### Health problems and grading of IAP

Health problems arises due to IAP was asked to the respondents and they were taken as layman reporting as their symptoms rather than and lab/clinical diagnosis. The degree of IAP was plotted based on the Warwick article (16) and focus group discussion in that community. Likewise, it is very difficult to measure the pollutants without any equipment in rural areas. Based on the study by Chowdhury et al. (18) and Dasugupta et al. in Bangladesh (19), the severity of IAP has been measured in three category measuring carbon monoxide (CO) and particulate matter_2.5–10_ (Table 1).

**Table 1:** **Average magnitude of pollutants within household**.

S No	Category	PM_2.5–10_ (mg m^−3^)	CO (PPM)
1	Smoking per episode within room	14 (20)	65.5
2	Biomass	263	62.6 (21)
3	Clean fuel	133	Few
4	Non-ventilation	0.45	11
5	Ventilation	0.10	1.6–4.4
6	Pollutant coverage (kitchen)	652	5.1–5.8
7	Pollutant coverage (outside the kitchen)	297	Few
6	Open fire space/traditional mud stove	0.45	4.4
7	Improve cooking stove	0.10	0.7

From the above table, the magnitude of IAP was categorized into no/mild, average, and severe (Table 2). It is the simplified measurement based on above articles where the application of equipment to measure is impossible.

**Table 2:** **Degree of air pollution from Table 1**.

Mild/no IAP	Average IAP	Severe IAP
Separate kitchen	Separate kitchen	No separate kitchen
At least 1 window or ventilation	No window/ventilation	No window/ventilation
Improve cooking stove	Improve cooking stove	Traditional mud stove
No Smoke within family	No smoke within family	Smoking person in family
Alternative using biomass	Always using biomass	Always using biomass

Furthermore, the category of the IAP was merged into two categories such as mild/no IAP to IAP (No) and average and severe IAP to IAP (Yes) due small or number of cell value during classification.

### Assessment of health status

The health problems were asked to the respondents as like Table 3.

**Table 3:** **Assessment of health status**.

S No	Conditions	Response
1	Do you have health problems since you are involving long time in your kitchen?	Yes
		No
2	How many times did you suffer since 1 year?	………
3	If yes, what kinds of health problems do you have? (multiple response)	Difficulty in breathing
		Dry cough
		Productive cough
		Tearing of eyes
		Itching of skin
		Headache
		Vertigo
		Others………
4	Are those problems recurrent?	Yes
		No
5	Do your kids (<5 years suffering from ARI since 1 year?	Yes
		No
6	How many times did he/she attack since last year?	………
7	Where did you treat?	Home remedy
		Hospitals and health centers
8	How much did you pay (in Nrs) for the treatment in last year for ARI to your children?	………

### Ethical consents

Verbal consent was taken from each participant who involved in the study and also the written permission was taken from authority of BP Koirala Institute of Health Science Dharan Nepal.

### Data entry and analysis

Data were entered into Microsoft Excel and exported to SPSS 20.0. Logistic regression and odds ratio were applied to show the association in different variables. Some variables were merged who had small value during the statistical analysis. Education category, type of houses, and stove used during cooking had merged into two categories from three categories during multivariate analysis. The problems associated with IAP were found by verbal reporting during research. Data quality was maintained by pre-test of questionnaire, training of the field researchers, data verification, and cleansing in both excel and SPSS formats.

## Results

The study area was rural area of Sunsari District in south part. Most (81.5%) household were under the poverty level (<1$ income/day). Except the caste (demographical variable) and separate kitchen (IAP factor), all variables were statistically significant (Table 4).

**Table 4:** **Descriptive findings from study (N = 157)**.

Characteristics	Variables	Category	Grading IAP ~ No. (%)			*p* Value
			No/mild IAP (%)	Average IAP (%)	Severe IAP (%)	
Demographic variables	Education	Illiterate	3 (23.1)	8 (61.5)	2 (15.4)	<0.001
		Primary	7 (14.6)	33 (68.8)	8 (16.7)	
		Secondary	7 (15.2)	23 (50)	16 (34.8)	
		Higher education	2 (4.0)	18 (36.0)	30 (60.0)	
	Geographical location	Rural	6 (4.8)	70 (55.6)	50 (39.7)	<0.001
		Semi urban	13 (41.9)	12 (38.7)	6 (19.4)	
	Caste	DAG	1 (7.1)	8 (57.1)	5 (35.7)	0.82
		Non-DAG	18 (12.6)	74 (51.7)	51 (35.7)	
	Per capita income per month	Up to 100$	13 (10.1)	64 (49.6)	52 (40.3)	0.05
		100–200$	5 (19.2)	17 (65.4)	4 (15.4)	
		>200$	1 (50.0)	1 (50.0)	0 (0.0)	
	Having own cultivating land	No	11 (11.3)	58 (59.8)	28 (28.9)	0.04
		Yes	8 (13.3)	24 (40.0)	28 (46.7)	
	Housing category	Dwelling and risk	12 (40)	16 (53.3)	2 (6.7)	<0.001
		Semi dwelling	2 (14.3)	8 (57.1)	4 (28.6)	
		Concrete/safe	5 (4.4)	58 (51.3)	50 (44.2)	
Factors related to IAP	Smoking behavior	No	18 (19.4)	73 (78.5)	2 (2.2)	<0.001
		Yes	1 (1.6)	9 (14.1)	54 (84.4)	
	Separate kitchen	No	4 (11.4)	15 (42.9)	16 (45.7)	0.35
		Yes	15 (12.3)	67 (54.9)	40 (32.8)	
	Cooking fuel	Biomass	9 (6.9)	68 (51.9)	54 (41.2)	<0.001
		No biomass	10 (38.5)	14 (53.8)	2 (7.7)	
	Ventilation in kitchen	No	5 (3.8)	71 (54.6)	54 (41.5)	<0.001
		Yes	14 (51.9)	11 (40.7)	2 (7.4)	
	Stove used during cooking	Traditional mud	2 (1.8)	58 (52.3)	51 (45.9)	<0.001
		Improved cooking	4 (20.0)	14 (70.0)	2 (10.0)	
		Electric/gas	13 (50.0)	10 (38.5)	3 (11.5)	
	Health problems	Yes	1 (0.9)	75 (54.7)	62 (44.4)	<0.001
		No	9 (45.0)	9 (45.0)	1 (10.0)	

Bivariate and multivariate analysis was performed to show the association between some variables with the health problems. Table 5 shows the odds ratio before and after adjustment where traditional mud stove (8.6; 3.0–24.7) and use of biomass (2.8; 0.4–16.3) in 95% CI were more risk than other variables.

**Table 5:** **Comparison of risk by odds ratio associated health problems on logistic regression**.

Characteristic	Categories	Health problems ~ No. (%)		OR (95% CI)	Adjusted OR (95% CI)
		No	Yes	
Geographical distribution	Semi urban	18 (58.1)	13 (41.9)	1	1
	Rural	22 (17.5)	104 (82.2)	6.5 (2.8–15.2)**	2.2 (0.3–14.4)
House type	Semi dwelling	20 (45.5)	24 (54.5)	1	1
	Dwelling and risk	20 (17.7)	93 (82.3)	3.8 (1.8–8.3)**	0.6 (0.1–2.3)
Caste	Non-DAG	38 (26.6)	105 (73.4)	1	1
	DAG	2 (14.3)	12 (85.7)	2.1 (0.4–10.1)	0.4 (0.08–2.7)
Education	Literate	34 (23.6)	110 (76.4)	1	1
	Illiterate	6 (42.2)	7 (53.8)	0.3 (0.1–1.1)	1.3 (0.2–8.6)
Smoking member in family	No	31 (33.3)	62 (66.7)	1	1
	Yes	9 (14.1)	55 (85.9)	3.0 (1.3–6.9)**	2.1 (0.7–5.8)
Separate kitchen	Yes	32 (26.2)	90 (73.8)	1	1
	No	8 (22.9)	27 (77.1)	1.2 (0.4–2.9)	0.6 (0.1–2.1)
Use of biomass	No	17 (65.4)	9 (34.6)	1	1
	Yes	23 (17.6)	108 (82.4)	8.8 (3.5–22.3)**	2.8 (0.4–16.3)
Ventilation in kitchen	Yes	21 (16.2)	109 (83.8)	1	1
	No	19 (70.4)	8 (29.6)	0.08 (0.03–0.2)**	0.2 (0.08–0.9)*
Type of stove	Improve cooking	28 (60.9)	18 (39.1)	1	1
	Traditional mud	12 (10.8)	99 (89.2)	12.8 (5.5–29.7)**	8.6 (3.0–24.7)**

*The adjusted variables were geographical distribution, house type, per capita income smoking, types of kitchen fuel, stove type, ventilation, and health problems by binary logistic regression. 1 is reference value*.

*p < 0.05, **p < 0.001.

**Figure 1:**
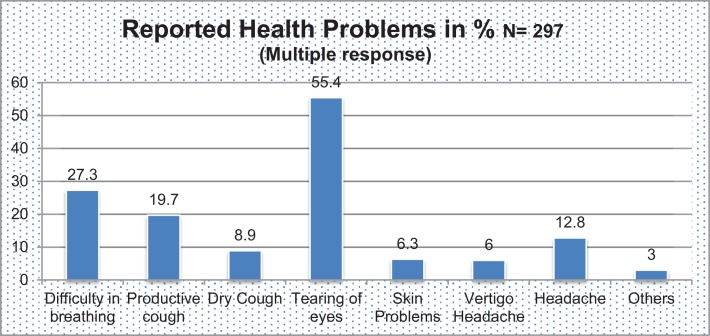
**Reported health problems due to indoor air pollution**.

In Table 6, there are indirect effects of the IAP. The treatment cost per household is three folder (301$ vs. 893$) higher in no/mild IAP than severe IAP. Similarly, average episode of the illness of child per year is almost double and average episode of illness adult is not high in severe IAP. Figure 1 shows the category of illness and it reveals 55.4% had tearing of eyes where as 6% reported vertigo since last year 2013.

**Table 6:** **Multiple effects associated with IAP**.

Level of indoor air pollution	Treatment expenditure in Rs/year/child	Average killing hour in kitchen/day	Average episode of illness of adult/year	Average episode of illness of child/year
No/mild IAP	301	3	2.1	4.3
Average IAP	465	3.5	2.6	4.6
Severe IAP	893	5.2	3.4	7.8

## Discussion

This study was carried out to explore the multiple effects of IAP in rural Terai area of Nepal. Before this study, some studies were conducted in hill and mountain area. Our study was simply designed but represents the global rural area. In other words, it is the simplest approach to find out the different magnitude of IAP in rural setting. Even it is useful to reduce the burden of IAP intervention strategy in small scale from the community level like during the construction of home and pollution free kitchen. The general illness was assessed that represents the morbidity pattern in the community without clinical and laboratory investigation. It is the significant achievement of the study. Poverty related factors like per capita income, education, housing condition, etc., are consistently associated with IAP. Use of traditional mud stove and unrefined biomass is the major risk factors for health problems associated with IAP (Table 5). Since, respiratory illnesses are the major causes of morbidity and mortality globally and in Nepal also (15, 22).

The poor health outcomes of respondents and their children were associated IAP in our findings (Table 3; Figure 1). Similar results were found with other related studies. A qualitative study conducted in rural area of Lalitpur district of Nepal showed ARI was one of the most prevalent and common where there was hazy and severe indoor air smoke (23). The prevalence of respiratory illnesses and symptoms were considerably higher in those living in mud and brick houses compared with concrete houses and prevalence was also higher in those living on hills and in rural areas compared with flatland and urban areas (24). It is similar findings to our study that there is statistically significant housing type and IAP. ARI increased from 0.03 to 0.56 per child when exposure level increased from 0.09 hour category to 4+ hours category, respectively, in poor communities (25). The prevalence of productive cough was 19.7% tearing of eyes is 55.4%, difficulty on breathing is 27.3%, asthma is 8.9%, skin problems 6.3%, vertigo 9.4%, and headache 12.8% within cooking member of family who are involving >5 years in kitchen; (25, 26) similar results with our study.

In global context, there is strong evidence of an association with use of solid kitchen fuels and acute lower respiratory infections (ALRIs) in children aged <5 years in comparative health risk assessed by WHO in developing countries (27). The occurrence of associated diseases, like acute upper and lower respiratory infections, COPD, asthma, perinatal mortality, pulmonary tuberculosis, LBW, eye irritation and cataract, etc., in Uttar Pradesh, India to the women who were responsible in cooking through household biomass (28). Solid fuel smoke like coal, firewood, steam of trees, etc., possess the majority of the toxins found in tobacco smoke and has also been associated with a variety of diseases, such as COPD in women, ARI in children, and lung cancer in women in Mexico (29). Exposure to solid fuel smoke is consistently associated with COPD and chronic bronchitis in developing countries; especially Africa continents (30). Child ALRIs, LBW, stillbirth, preterm birth, stunting, and all-cause mortality in children and equally risk of COPD, tuberculosis, and even lungs cancer to their mothers were heavily associated with IAP in China (31). Continued exposure to smoke from traditional fuels has been shown to cause acute respiratory illnesses (ARI), COPDs, lung cancer, blindness, and TB in another study in China (32).

In Nepal’s hill areas, women’s total recorded work time was found to be between 150 and 180% of that of men, out of which 40% was spent on fuel collection alone (33). For the sites where deforestation was greatest, the time required to collect a standard load of fuel wood was 75% higher than it was where deforestation was low, which translated to a 45% increase in time spent for fuel wood collection (34). Additionally, spending hours for cooking in hazardous conditions in inefficient stoves result rise to eye infections and other respiratory problems. Similarly, women had to spend considerable amount of time in collecting kitchen fuel thereby reducing the time available for education and income generating activities (35). Our surplus findings show that the treatment cost is about three folder higher, average episodes being ill to children and adult, more than two times higher to spend time in kitchen in severe IAP than no/mild IAP. These associated findings show the indirect impact of IAP.

There are some limitations of this study, where one is the precision of IAP and another is confirmation of the disease or illness because there are no use of scientific equipment to measure the pollutant level and clinical diagnosis of disease/illness. Another, the severity of IAP was based on CO and PM though other pollutants were not taken into account. The level of precision was 0.07 due to errors in some samples. The condition of biomass (wet/dry), type of biomass, other human behaviors, etc., could not to include in the standard and need to verify by scientific equipment.

## Conclusion

So in developing countries, most of the population is in rural area, they are suffering from IAP directly or indirectly especially women and children. There are some scientific standard of good housing (36), but they are not practical to improve in rural context. So, practical standards could be helpful to reduce the magnitude of IAP and reform the housing standard in those rural areas. This approach will not be gold standard to measure the indoor pollutants but screening process to measure the nature and magnitude of the pollution level by specific biomasses that are hazardous. Some effective interventions are suggested to reduce the severe level of IAP in community.

## Conflict of Interest Statement

The authors declare that the research was conducted in the absence of any commercial or financial relationships that could be construed as a potential conflict of interest.

## Abbreviations

ALRI, acute lower respiratory infection; ARI, acute respiratory infection; CI, confidence interval; COPD, chronic obstructive pulmonary disease; CD, cluster of difference; DAG, disadvantage group; FGD, focus group discussion; IPAID, Institute for Poverty Alleviation and International Development; WHO, World Health Organization.
